# Predation Risk within Fishing Gear and Implications for South Australian Rock Lobster Fisheries

**DOI:** 10.1371/journal.pone.0139816

**Published:** 2015-10-21

**Authors:** Felipe Briceño, Adrian Joseph Linnane, Juan Carlos Quiroz, Caleb Gardner, Gretta Tatyana Pecl

**Affiliations:** 1 Institute for Marine and Antarctic Studies (IMAS), University of Tasmania, Hobart, Tasmania, Australia; 2 South Australian Research and Development Institute (Aquatic Sciences), Adelaide, South Australia, Australia; Seagrass Ecosystem Research Group, Swansea University, UNITED KINGDOM

## Abstract

Depredation of southern rock lobster (*Jasus edwardsii*) within fishing gear by the Maori octopus (*Pinnoctopus cordiformis*) has economic and ecological impacts on valuable fisheries in South Australia. In addition, depredation rates can be highly variable resulting in uncertainties for the fishery. We examined how in-pot lobster predation was influenced by factors such as lobster size and sex, season, fishing zone, and catch rate. Using mixed modelling techniques, we found that in-pot predation risk increased with lobster size and was higher for male lobsters. In addition, the effect of catch rate of lobsters on predation risk by octopus differed among fishing zones. There was both a seasonal and a spatial component to octopus predation, with an increased risk within discrete fishing grounds in South Australia at certain times of the year. Information about predation within lobster gear can assist fishery management decision-making, potentially leading to significant reduction in economic losses to the fishery.

## Introduction

Predation plays an integral role in marine ecosystems influencing the structure and dynamics of ecological communities, with direct effects on prey populations via density-dependent mechanisms (e.g. predator responses [1,2]), as well as indirect effects through altering prey behaviour and physiology ([3]). In fisheries, predation is considered as a pervasive but ephemeral feature [4] and represents a large source of fish mortality, which in many cases, exceeds fishing mortality [5]. Additionally, predators are able to interact directly with fisheries by preying upon target species caught within fishing gear, mortality known as ‘depredation’ [6]. Information about predator–fishery interactions have been mostly reported from top predator depredation in long-line fisheries [7–9]. However, depredation can occur in a variety of fishery systems, including trap-based fisheries for lobster and crab where teleosts (e.g. conger eels, [10]), elasmobranchs (e.g. catsharks,[11]), and cephalopods (e.g. octopus, [12,13]) are common middle-trophic predators within fishing gear. In contrast to depredation from top predators, the knowledge about middle-trophic predators interacting with crustacean fisheries has received less attention, despite substantial economic ([13]) and ecological implications ([11]). Octopus depredation has been the subject of research on crab and lobster fisheries from the beginning of last century ([14]), driven by the value of the loss of product as per this research on the southern rock lobster fishery in South Australia ([13,15–17]).

Most octopuses are generalist predators, displaying an opportunistic feeding behaviour strongly linked to prey abundance and environmental conditions [18]. Adult lobsters and crabs caught in traps are unable to escape from foraging octopuses which are able to easily enter traps and kill individuals before they are harvested by fishers. Depredation risk by octopus is difficult to predict given strong inter-annual variation in octopus abundance, particularly within areas that experience extreme temperature variation [14,19]. Additionally, crustacean fisheries can be affected by the consumption of bait by octopus in lobster traps because this prevents subsequent lobster capture [20,21]. Despite the economic impacts of lobster mortality and bait consumption, effects of octopus predation have been underestimated in many fisheries [21] and its quantification and incorporation into stock assessments of lobster fisheries is spatially and temporally limited [17].

### The octopus–rock-lobster fishery interaction in South Australia

The South Australian rock lobster (*Jasus edwardsii*) fishery (SARLF) has a gross value of $86.1 million from 1,552 tonnes of production (2012/2013) [22]. The SARLF is divided into two management zones–the northern and southern zones–with the latter being the most productive zone [23–25]. Approximately 98% of total in-pot lobster mortality in the SARLF is due to predation by the Maori octopus (*Pinnoctopus cordiformis*, also known as *Octopus maorum* [26])[10], which is the largest octopod in Australasia [27]. Lobster mortality and octopus catch through time are highly correlated in the SARLF [24,25] suggesting that in-pot octopus predation is influenced by trends in octopus abundance. Additionally, octopus depredation risk decreases with depth so that there is greater impact in inshore SARLF areas (<60 m depth) [13]. Between 1993 and 2013, a total of 3,289,538 lobsters were reported killed by octopus (<60 m) in the SARLF (average 164,000 per year; S1 Fig) with large spatial and temporal variation [24,25]. Additionally, although the current investigation concentrates on South Australia, the interaction between *P*. *cordiformis* and *J*. *edwardsii* within fishing gear is also known to lead to significant economic losses elsewhere including in Tasmania [15–17] and New Zealand [28].

**Fig 1:**
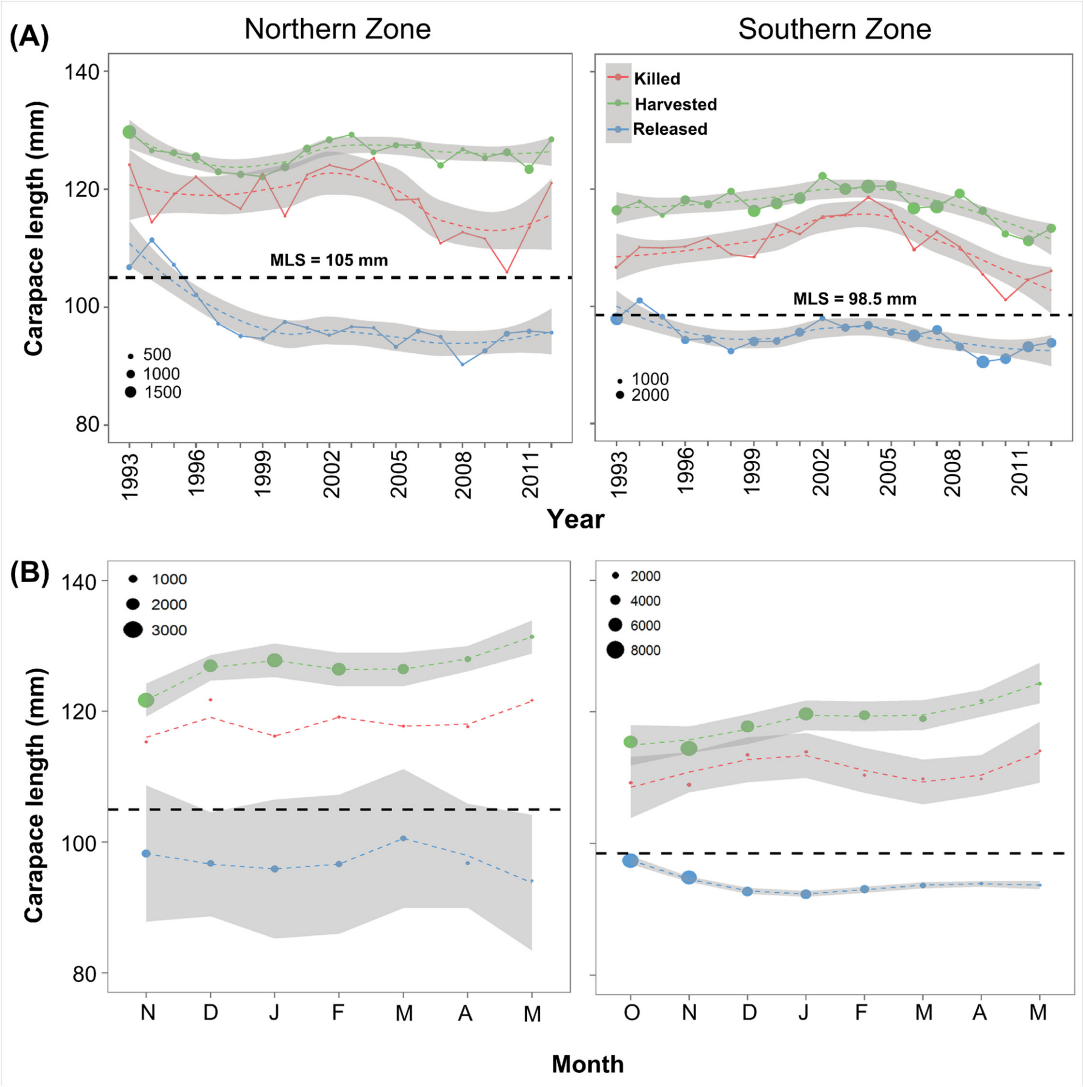
(A) Size time series for killed, harvested and released lobsters from the volunteer catch sampling program for the rock lobster fishery in the Northern Zone (NZ) and Southern Zone (SZ) of South Australia. (B) Lobster size distribution at a monthly scale for the same categories. Mean values are represented as circles with circle size representing the number of observations. Dashed lines represent the smoothing (polynomial), and the grey bands represent the confidence interval around the mean (mean + 1.96*sd). Horizontal dashed line represents the minimum legal size (MLS) for each zone.

Data on lobster size are collected through routine fisheries research programs and this has shown that the size of lobsters killed by in-pot predation has been decreasing over recent years and is now converging on the minimum legal size (MLS) for lobsters (Fig 1). It is uncertain how this trend may impact the fishery, although we note the current stock assessment model is length based (carapace length) and thus there is capacity to include changes in size-specific mortality [29] in assessments and harvest strategy evaluation. Consequently, a finer examination of key life history traits, such as size and sex are needed to understand octopus depredation in the SARLF. In addition, the nature of interactions between lobster catch and depredation within fishing gear warrants research because complex patterns have been observed elsewhere [10, 22, 23]. For example, a depensatory mortality mechanism was suggested to explain the inverse relationship between lobster mortality and lobster catches occurring homogenously across the stock assessment areas in Tasmania [15]. More recently, it’s been shown that this relationship is dynamic temporally and spatially [10]. Understanding predator–prey interactions in marine fisheries appears as a key component in the implementation of ecosystem-based management [5], with the identification of key trophic linkages resulting in a better capacity to model marine fish populations and food webs [2].

The present study examines how individual traits of *Jasus edwardsii*, such as body size and sex affect risk of in-pot predation, including whether relationships vary spatially. In addition, we examine how lobster catch rates affect predation risk at a daily scale. Here, lobster mortality is used as a proxy of ‘in-pot predation risk’ for the SARLF, and we provide insights about temporal and spatial components of octopus depredation that could be beneficial for improving fishery management.

## Methodology

### Southern rock lobster fishery in South Australia

The northern zone (NZ) and southern zone (SZ) fisheries in South Australia are managed using a combination of input and output controls [24,25], with data and assessment of these zones further subdivided into marine fishing areas (MFAs). Since 1993, the fishery has been controlled by annual total allowable commercial catches (TACCs), which apply separately across each zone and are divided proportionally among licence holders owning individual transferable quota units (ITQs) [24,25]. In 2013, the TACCs in the NZ and SZ were 345 tonnes and 1,250 tonnes, respectively [24,25]. The fishing season runs from November to May in the NZ, and from October to May in the SZ. Seasons for both zones are referred to here by start-of-season year [24,25]. The MLS in the NZ is 105 mm carapace length (CL), whereas in the SZ it is 98.5 mm CL [24,25]. Fishers in both zones may use up to a maximum of 100 pots [24,25].

### Fishery-dependent size sampling

Data on lobster size from 1993 and 2012 was used for this study obtained from a voluntary fishery-dependent sampling program. This program of voluntary catch sampling by commercial fishers and on-board observers has been implemented in the SRLF since 1991, which provides size measurements of legal and undersize lobsters, as well as the number killed through predation [24,25]. Fishers are encouraged to sample up to three pots per day, while observers sample all pots. Details on sampling effort are provided as supporting information (Table A in S1 Appendix). All escape gaps in catch sampling research pots are closed to increase catch of smaller lobsters. Fishers and observers are able to easily recognize in-pot octopus predation as lobsters are killed without damage to the exoskeleton, which appears to be ‘sucked clean’ [30,31] (S2 Fig).

**Fig 2:**
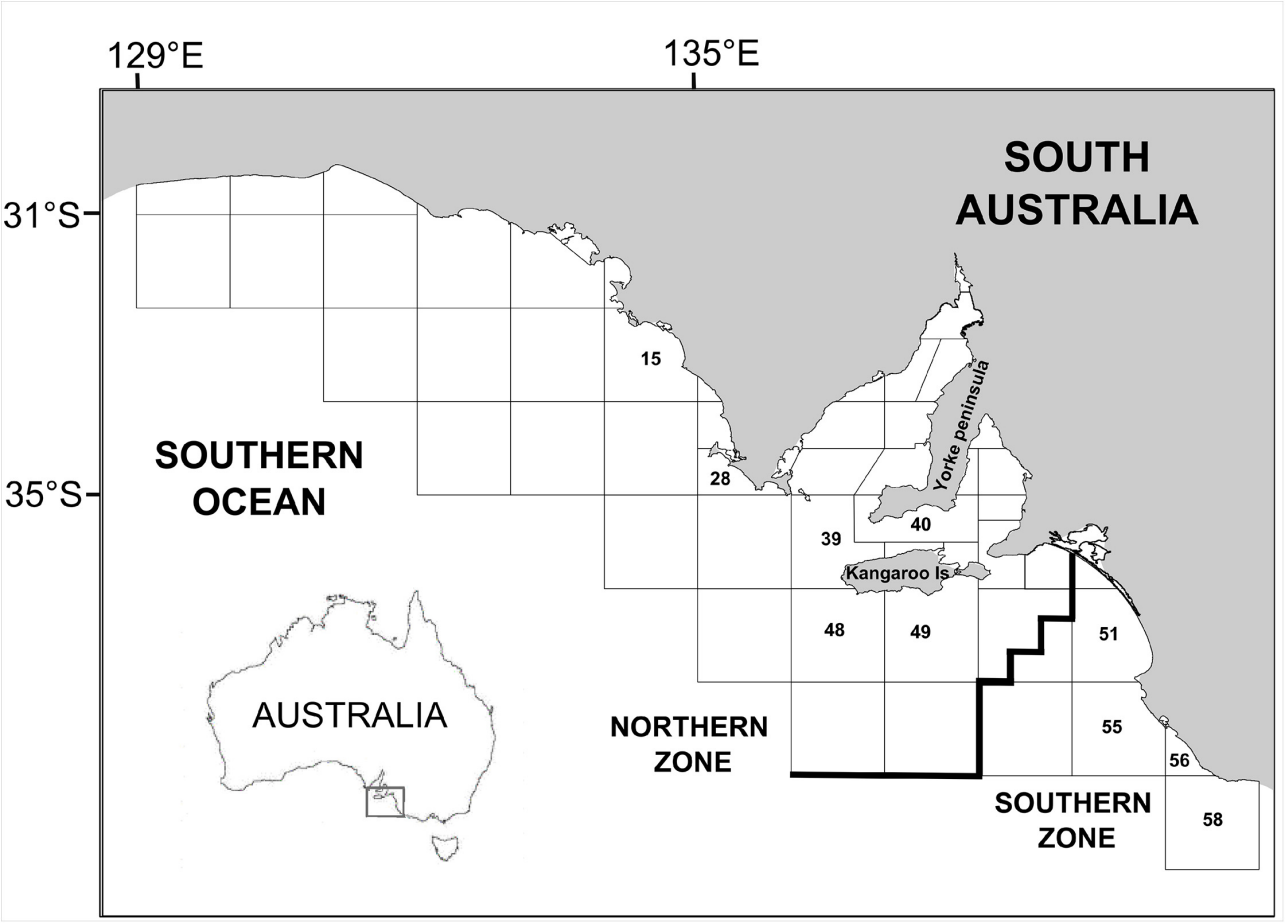
Fishing zones (northern and southern) with marine fishing areas (MFA) for the rock lobster fishery in South Australia. The MFAs used for this study are specified with numbers.

We used three categories of lobsters: retained or harvested (H), killed (K) and released (R) lobsters, with released lobsters being those that were undersize. Lobster carapace length (CL, mm) was recorded and referenced by day, depth and MFA. The following MFAs were used for each zone [24, 25]: MFA 15, 28, 39, 40, 48 and 49 in the NZ, and MFA 51, 55, 56 and 58 in the SZ (Fig 2). These MFAs reflect where >90% of the catch is taken annually [24, 25].

Around 90% of catch is taken in inshore areas (<60 m depth) [23], where in-pot octopus predation is higher than offshore areas [13]. Hence, data used for this study were also standardized by depth (≤60 m). The proportion of lobster killed was calculated as total number killed (K) from the total catch (H+R).

### Modelling

#### Testing dependency of life history traits and fishing zones

The relationship between the probability of lobster mortality and lobster size and sex was modelled using the spatial dependency with fishing zones as predictors. A total of 39,844 lobsters (67.63% female) were included in the analysis. Sample size was balanced at each temporal and spatial stratum resulting in a balanced design matrix (Table B in S1 Appendix), which reduces the risk of bias and ambiguity in linear predictors. Given that temporal changes in lobster size have been documented in both fishing zones (Fig 1), we developed a generalized linear mixed model (GLMM) including year as a random intercept, which was nested by zone to reduce the temporal and spatial variability of predictors. The model (Model 1) was selected from a total of five model candidates using as criteria the lowest log-likelihood value, Akaike information criteria (AIC), and degrees of freedom. Further details on model candidates are provided as supplementary information (Table A and Figure A in S2 Appendix). The binomial probability distribution was given by incorporating ‘killed’ or ‘live’ as a binary response, therefore the binomial GLMM (Model 1) was specified as:

Model1:P(killed)∼size+sex+zone,random=∼1|year(zone),

where *P* is the probability of lobster mortality at given lobster size, sex and fishing zone.

#### Testing density-dependency with lobster catches

A second model (Model 2) was constructed to examine whether the probability of lobster mortality depended on lobster CPUE. Daily commercial lobster catch (i.e. logbook data) from the selected MFAs was used as an alternative data source. Calculating lobster CPUE from the voluntary program can lead to problems with data dependency (e.g. circularity), thus we utilized commercial CPUE into these models. This dataset was matched with size data by date and depth (≤60 m), which resulted in a total of 35,724 analysed observations. A binomial GLMM was applied using as fixed predictors size, sex and the interaction between lobster CPUE and MFA. A total of five model candidates were tested, and the selected model was chosen following the same criteria as specified in Model 1. Model candidates and criteria used are provided as supporting information (Table B and Figure B in S2 Appendix). The interaction lobster CPUE:MFA was included given the high spatial dependency of lobster catch rate. The temporal variability in lobster catch rate was modelled by including the terms ‘year’ and ‘month’ as random factors within the GLMM. This resulted in the following binomial model:

Model2:P(killed)∼size+sex+CPUE:MFA,random=∼1|(year+month),

where *P* is the probability of lobster mortality at given lobster size and sex and CPUE:MFA is the interaction between lobster CPUE and marine fishing zones (MFA). Together with the forward step applied to define the GLMM, we tested GLM models for lineal predictors exclusively. GLM models showed lower goodness of fit compared with GLMM (Figure B in S2 Appendix). In addition, we further examined temporal components in octopus depredation at inter- and intra-annual (e.g. within fishing season) scales by including year and month as fixed factors in Model 2. All analyses were performed in R using package ‘lme4’ and ‘MASS’[32].

## Results

### In-pot predation risk and lobster life history traits

The probability of lobster mortality was dependent upon lobster size, with larger lobsters more likely to be killed (df = 1, F = 28.96, p<0.001) (Table 1). In addition, predation risk was affected by lobster sex, with more males killed than females (df = 1, F = 8.25, p<0.01). In-pot predation risk differed among zones, being higher in the SZ than the NZ (df = 1, F = 34.34, p<0.001).

**Table 1:** Parameter estimates from GLMM modelling of the effect of lobster size, sex, and fishing zone on the probability of lobster mortality (Model 1).

**Random effects**				
**Parameter**	**Variance**	**Std. Dev**		
Zone: Year (intercept)	1.850e-02	0.136		
Year (Intercept)	0.015	0.12		
**Fixed effects**				
**Parameter**	**Value**	**SE**	**z-value**	**p-value**
Intercept	-4.61	0.190	-24.23	<0.0001
Size	0.09	0.016	5.77	<0.0001
Sex	0.29	0.054	2.69	<0.01
Zone	0.36	0.063	7.35	<0.0001

### In-pot predation risk and lobster CPUE

The effect of lobster catch rate or CPUE on in-pot predation risk varied among MFAs, with significant effects in MFAs 15, 28, 39 from the NZ and MFA 55 in the SZ (p<0.005). The effect of CPUE also varied in direction between MFAs with higher predation risk at low lobster CPUE in northern MFAs 15, 28 and 39 but high predation risk at high lobster CPUE in the SZ MFA 55 (Table 2). Moreover, the strength of this relationship varied between MFAs with the highest coefficients in MFA 15 and the lowest in MFA 55. Additionally, the random factors ‘year’ and ‘month’ varied 10.6% and 15.4% o respectively, demonstrating that in-pot predation risk varied more at an intra-annual scale (fishing season) than an inter-annual scale.

**Table 2:** Parameter estimates from GLMM modelling of the effect of lobster size, sex, and the interaction between lobster catch rate (CPUE): MFA on the probability of lobster mortality (Model 2).

**Random effects**				
**Parameter**	**Variance**	**Std. Dev**		
Year (intercept)	0.011	0.106		
Month (intercept)	0.024	0.154		
**Fixed effects**				
**Parameter**	**Value**	**SE**	**z-value**	**p-value**
(Intercept)	-4.05	0.188	-21.50	<0.0001
Size	0.07	0.014	4.74	<0.0001
Sex	0.16	0.057	2.84	<0.01
CPUE: MFA 15	-1.05	0.311	-3.40	<0.001
CPUE: MFA 28	-0.40	0.133	-2.95	<0.01
CPUE: MFA 39	-0.42	0.113	-3.70	<0.001
CPUE: MFA 40	-0.13	0.180	-0.74	0.46
CPUE: MFA 48	-0.17	0.181	-0.93	0.35
CPUE: MFA 49	0.01	0.124	0.10	0.92
CPUE:MFA 51	0.18	0.128	1.41	0.16
CPUE: MFA 55	0.14	0.064	2.20	<0.05
CPUE: MFA 56	0.08	0.052	1.59	0.11
CPUE: MFA 58	-0.05	0.064	-0.74	0.46

**Fig 3:**
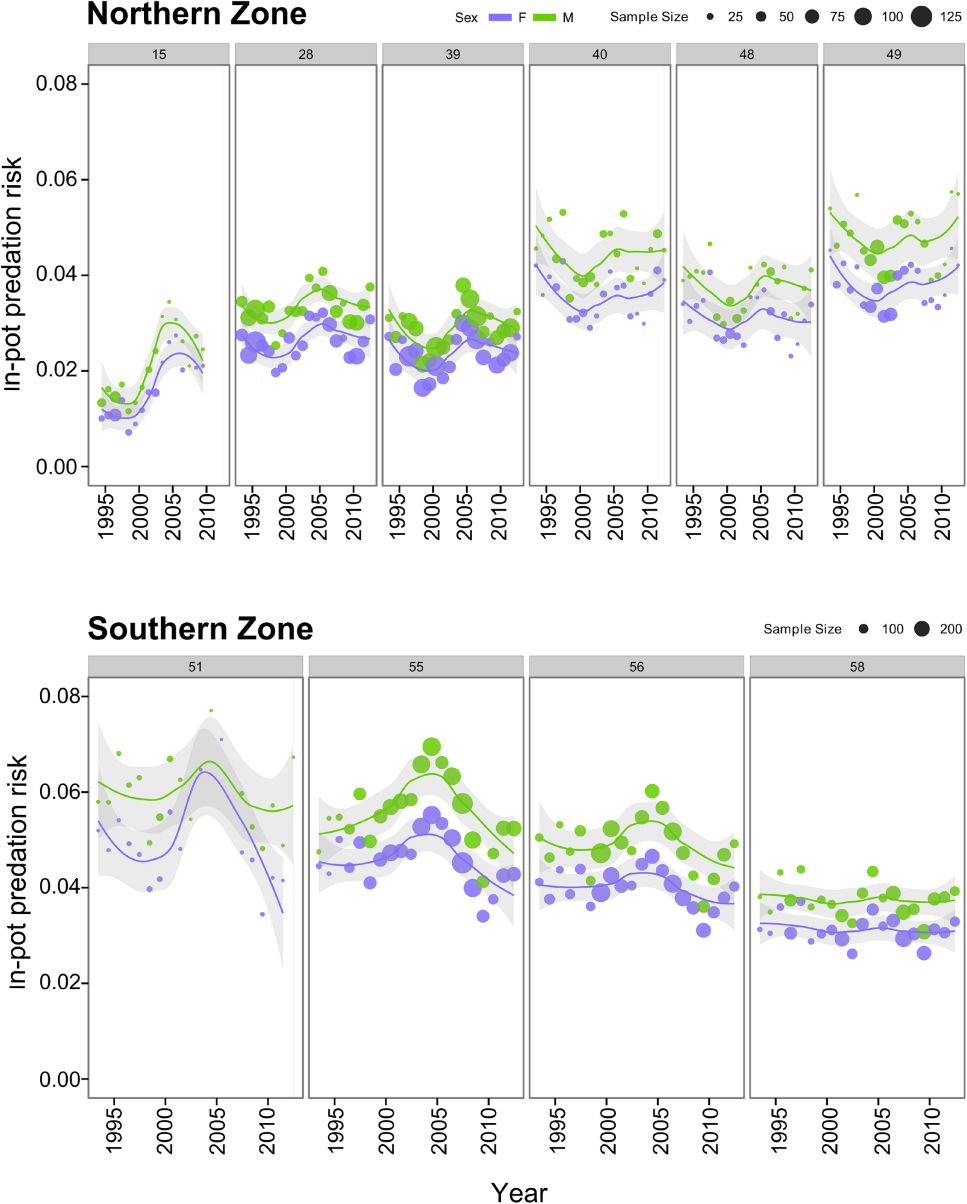
In-pot predation risk between 1993 and 2012 from fitted values (Model 2) across selected marine fishing areas from northern and southern zone in the rock lobster fishery of South Australia.

Fitted values (Model 2) across years showed spatial variation within in-pot predation with elevated levels of predation risk in specific sites off Kangaroo Island (MFA 48 and 49) and the Yorke Peninsula (MFA 40) (Fig 3). Mean fitted values and coefficient of variation (CV%) were 3.02% (CV = 32.7%) for NZ and 4.46% (32.39%) for SZ. Mean fitted values of in-pot predation risk among NZ MFAs were: MFA 49 (4.05%) ~ MFA 40 (4.01%) > MFA 48 (3.27%) > MFA 28 (2.94%) > MFA 39 (2.61%) > MFA 15 (1.56%). The coefficient of variation (%) of these values also included a spatial component with larger fluctuation among years towards northern areas (e.g. MFA 15, 45.86%). In-pot predation risk for SZ MFAs followed this order: MFA 51 (5.34%) > MFA 55 (5.14%) > MFA 56 (4.45%) > MFA 58 (3.39%). Conversely, inter-annual variability among these areas was more elevated in MFAs 51 and 55 than MFAs 56 and 58.

### Seasonal trends of in-pot predation risk

**Fig 4:**
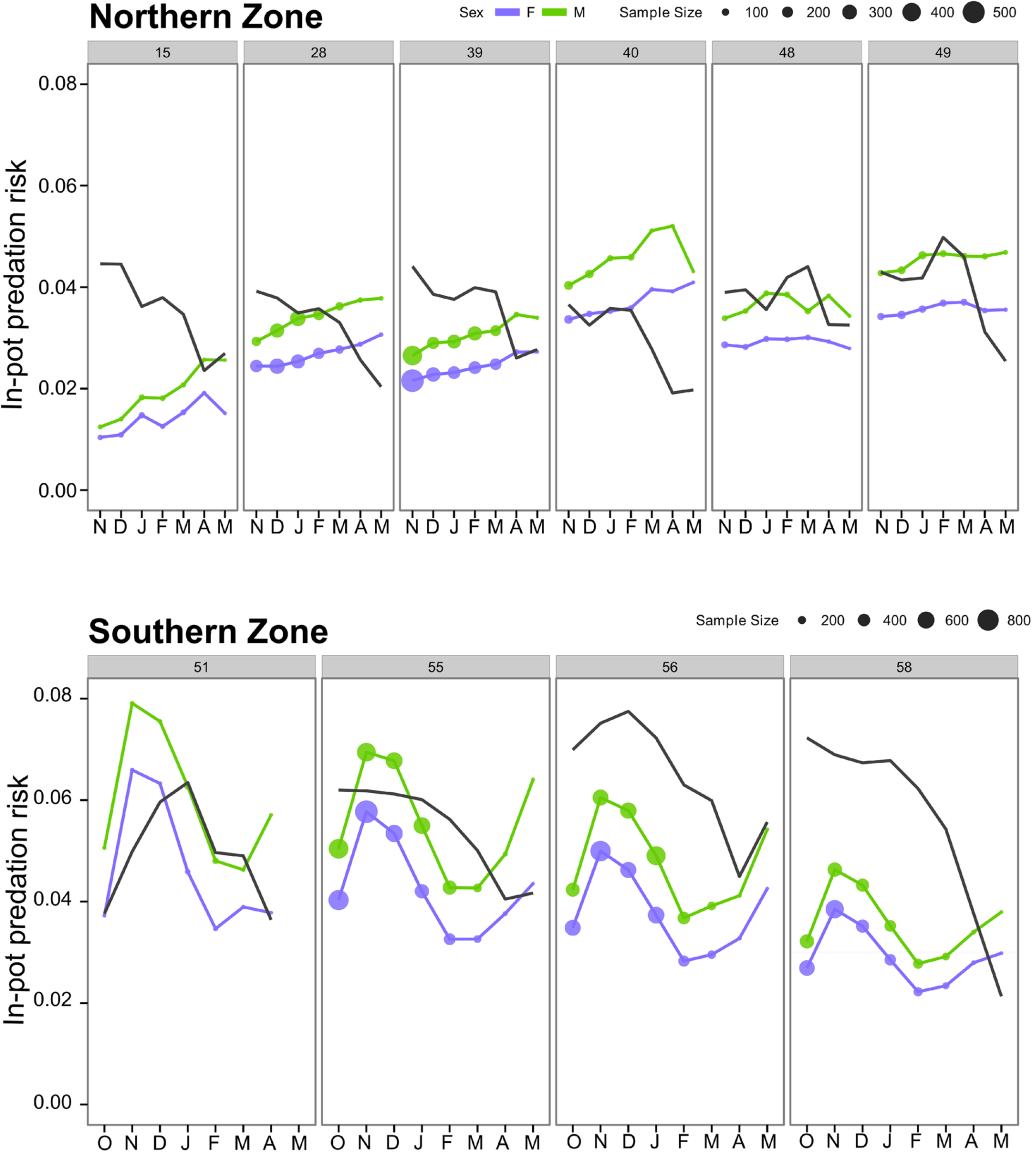
In-pot predation risk and lobster CPUE (black line) throughout the fishing season for selected marine fishing areas from northern and southern zone in the rock lobster fishery of South Australia. CPUE was scaled to maximum by zone*sex (max NZ = 1.57 lobster/pot, max SZ = 2.02 lobster/pot).

An extended version of Model 2 was performed using month as predictor to examine in-pot predation risk across the fishing season (Fig 4). We found that predation risk steadily increased across the fishing season in the NZ, with the highest levels close to the end of the season in April and May when catch rate and catch were at their minimum levels (Fig 4). While predation risk was relatively stable across fishing season in MFA 48 and 49, a higher intra-annual variability was found in MFA 15. The trends in predation risk were broadly similar across all MFAs in the SZ, reaching maximum levels in November before declining over the next three months and rising at the end of the fishing season in April and May.

## Discussion

In this study, we investigated the effect of lobster life history traits (size and sex) and lobster CPUE on the probability of lobster mortality by octopus depredation in the South Australian Rock Lobster Fishery (SARLF). We found that the predation risk of lobsters was higher for males and increased with size, particularly in the southern zone. Likewise, the relationship between predation risk and lobster CPUE varied at the MFA level, which would suggest different density-dependent mechanisms are affecting the octopus–SARLF interaction in different locations. The relationships among size, sex and density dependencies in lobster mortality in the SARLF were explored, integrating information on fishing factors, lobster ecology and octopus predatory behaviour.

### Size and sex dependency of in-pot predation risk

Octopus depredation was higher for larger individuals with males more likely to be killed than females as per results from a short-term study undertaken in the SZ during the 2000–2001 fishing season[13]. Research from a nearby fishery in Tasmania for the same species failed to identify sex-dependant mortality by octopus [33], showing broader scale spatial differences in the pattern of octopus predation. Size- and sex-dependent mortality may mirror the catchability and the selectivity of the SARLF. In the Tasmanian rock lobster fishery, larger lobsters were found to have higher catchability than smaller lobsters, and males occur in the pot more often than females due to behavioural interactions (e.g. a dominance hierarchy of agonistic interactions among different-sized individuals) [34,35].

### Density-dependence of in-pot predation risk

The effect of lobster CPUE on octopus-related mortality in the SARLF differed spatially across MFAs, similar to that which occurred in Tasmania [17]. We also found that the seasonal component of fitted values showed higher variation from the predictor ‘month’ compared with the predictor ‘year’. This suggests that there is a predictable seasonal pattern in mortality risk throughout the fishing season and that this pattern is more important than variation from year to year.

Lobster catchability highly depends on feeding behaviour, which is seasonally modified by key physiological and ecological processes such as moulting and mating [36]. Seasonality in in-predation risk could be also related to changes in octopus abundance within the fishing ground as suggested in similar studies. Additionally, seasonality of in-pot predation risk could be associated with seasonal changes in octopus abundance on the lobster fishing grounds as reported in some studies [13]. For example, coastal octopuses such as *P*. *cordiformis* perform seasonal migrations to deeper water for reproduction which would lead to changes in predatory pressure on lobster.

An inverse correlation between lobster catch rate and lobster mortality was found in most areas of the NZ. This suggests that a ‘depensatory mortality’ mechanism may be operating, arising from predator saturation (type II functional response, [1]) or/and from predator avoidance tactics (e.g. ‘group-defence’ effect)[15,37]. However, depensation in octopus-related mortality was also found here to be affected by lobster size. Catches in the NZ have high variation in lobster size, with larger average sizes towards northern MFAs [38]. This pattern in size of catch may affect octopus predatory behaviour by access to large animals with a low number of individuals per pot (0.02–0.04 lobsters per pot). This is consistent with a recent experimental tank study where the number of attacks by octopus was higher on single lobsters than lobster in groups [39], likely due to group anti-predator strategies [37]. Moreover, the octopus hunting strategy is more effective where lobsters are in reduced spaces [40,41] such as a pot than in the open, which is also known to affect lobster distribution in the wild [42,43]. Previous investigations have shown a flexible activity pattern of *P*. *cordiformis* within lobster pots under experimental conditions in tanks [44], although further studies looking at how lobster catchability can be modified by octopus presence as well as others factors involved in killing success deserves attention. For example, the effect of presence of conspecifics of varying size or predators other than octopus on the success of octopus in killing lobsters within traps may be important.

Octopus predation in the SZ followed a different pattern to the NZ as in-pot predation risk increased proportionally with lobster catch rates in MFA 55. MFA 51 and 55 in the northern region of SZ are characterized by catches of small numbers of larger sized lobsters in comparison with MFAs in the southern region (MFA 56 and 58) [45] and this may have contributed to the spatial patterns detected in octopus predation. A direct relationship between number of prey consumed and prey density, defined as functional response type I [1], assumes that the time spent by predator handling and processing the food is negligible, or that the consumption of food does not interfere with predator food searching. Suitable experiments testing functional and numerical responses in predator–prey interactions are needed in marine fisheries [2]. Further studies examining predatory mechanism in octopus depredation are relevant in this particular area.

Overall, our findings reveal a significant spatial component in octopus depredation within the SARLF. Such spatial variability in predation risk could be attributable to differences among fishing zones in terms of lobster life history (e.g. growth and maturity[46]), oceanographic conditions (e.g. upwelling events, [47]) as well as habitat type and depth (e.g. [46]). For example, the spatial heterogeneity in growth of *J*. *edwardsii* in South Australia is suggested to be a density-dependent process [46], with higher densities and therefore slower growth, in the SZ compared to the NZ. Higher densities in the SZ appear to be associated with higher levels of puerulus settlement which ultimately translates into higher levels of fishery recruitment compared to other regions in South Australia. In addition, lobster habitat in the SZ is more continuous, consisting mainly of bryozoan or aeolianite limestone reef, compared with the more discrete and isolated granite outposts found in the NZ ([38]). Habitat complexity plays a crucial role in the different ecological traits of lobsters particularly under predation risk (e.g. [48]). Additionally, studies have demonstrated that octopus presence can strongly alter distribution and habitat selection in lobsters (e.g. *Panulirus argus* [42,43]). Finally, spatial variation in prey density, refuge availability and environmental conditions (e.g. temperature) can strongly constrain octopus foraging ecology and demographic traits (e.g. population size [49]).

### Seasonal models of in-pot predation risk

#### Northern Zone

In-pot predation risk increased through the fishing season, reaching maximum levels in April and May when lobster catch rates and catches were lowest. Lobster catchability highly depends on the lobsters’ feeding behaviour, which is seasonally modified by moulting and mating [36]. The high predation-risk at the end of the season is expected to impact large males given their dominance in pots at this time [24]. Moreover, risk was lower at the beginning of the season which should reduce in-pot predation of spawning females, which are rarely caught after November [24]. Size of lobsters in catches and thus predation risk is affected by market dynamics, with fishers targeting less desirable large, typically male lobsters when supply levels are low such as during winter [50]. Depredation is thus another source of removal in addition to fishing that could modify reproductive behaviour as large males are suggested to control access to females in *J*. *edwardsii* [51].

#### Southern Zone

High predation risk occurred mainly in November–December, following the opening of the fishing season, plus later in April–May. This may impact on different groups of lobsters given seasonal changes in size and sex catchability. Males moult at the start of the season and are under-represented in the catch until later in the season when catchability of females declines due to moulting and mating [25]. A sex ratio skewed towards females in catches in the first few months of the season increases their risk of predation including while ovigerous and possibly less mobile in January–February [25]. Impacts on females are clearly of interest due to the direct effect on egg production.

### Impacts and mitigation actions

This study provides information to broadly quantify losses of lobsters from depredation in the SARLF at MFA levels. Using mean values of in-pot predation risk by zone (Model 2) (NZ = 3.02%; SZ = 4.46%), the additional mortality from depredation in 2012 [24, 25] would approximate 10 tonnes in NZ (TACC = 345 tonnes) and 56 tonnes in SZ (TACC = 1250 tonnes). Assuming a price of AU$55/Kg [22], the financial loss would be AU$ 0.6 million in NZ and AU$ 3.08 million for the 2012 season. In-pot predation risk varies significantly at seasonal and MFAs scales so the economic impact will vary and will be far higher in some years. Furthermore, data used here excluded lobster mortality from offshore catches (>60 m), and did not include additional economic loss associated with bait consumption by octopus [44].

Despite considerable economic loss to rock lobster fisheries due to octopus depredation, interactions between octopus and pots are difficult to avoid and are therefore considered to be inevitable in this commercial fishery. Attempts to reduce mortality traditionally include hauling gear early in the morning but there has also been research on reducing mortality through gear modification. This research involved modifications to conventional pots to create two chambers [52]. These reduced depredation but were not adopted commercially due to the reduced catches of legal-sized lobsters [52].

The findings from this study could contribute to stock assessment of the SARLF. The current fishery model used to assess the performance of the fishery [29] is not only length based, but is also both spatially and temporally explicit. By identifying how lobster predation is impacted by size, sex, fishing zone and time period, annual estimates of lobster biomass can be considerably enhanced thus assisting sustainable management of this economically important fishery resource. Understanding these patterns could assist future discussions on changing any aspects of management of the fishery, especially if these involve shifting catch between areas or months.

## Supporting Information

S1 Appendix Sampling size. **Table A:** Mean and maximum number of pots sampled per day by year and zone used in this study to examine octopus depredation in the rock lobster (*Jasus edwardsii*) fishery in South Australia. **Table B**: Total lobsters sampled by year and fishing zone, including sex proportion, used in this study to examine octopus depredation in the rock lobster (Jasus edwardsii) fishery in South Australia.
(PDF)

S2 Appendix Model selection. **Table A:** Model candidates proposed to test dependency of size, sex and zone in lobster mortality by octopus depredation within rock lobster fishery (*Jasus edwardsii*) in South Australia. **Table B:** Model candidates proposed to test dependency of size, sex, MFA and lobster catch per unit effort (cpue) in lobster mortality by octopus depredation within rock lobster fishery (*Jasus edwardsii*) in South Australia. **Figure A:** Model selection criteria based on (a) Log-likehood (b) Akaike information criteria (AIC) used to define Model 1. **Figure B:** Model selection criteria based on (a) Log-likehood (b) Akaike information criteria (AIC) used to define Model 2.
(PDF)

S1 Fig Total lobsters killed by octopus within lobster pots between 1993 and 2011 in the northern (N) and southern (S) fishing zone of the rock lobster fishery (*Jasus edwardsii*) in South Australia. Dashed blue black lines represent the smoothing (polynomial), and the grey bands represent the confidence interval around the mean (sum+ 1.96*sd).
(TIF)

S2 Fig Lobster killed by an octopus found in lobster trap in Tasmania. The picture shows the characteristic pattern of most of the muscle and viscera removed by the octopus (Felipe Briceño, February 2012).
(TIF)
