# Supplementary material for: Predation Risk within Fishing Gear and Implications for South Australian Rock Lobster Fisheries
Source: PLoS One. 2015 Oct 21;10(10):e0139816. doi: 10.1371/journal.pone.0139816 (PMC4619570; doi:10.1371/journal.pone.0139816)
Supplement: S1 Appendix — Table A: Mean and maximum number of pots sampled per day by year and zone used in this study to examine octopus depredation in the rock lobster (Jasus edwardsii) fishery in South Australia. Table B: Total lobsters sampled by year and fishing zone, including sex proportion, used in this study to examine octopus depredation in the rock lobster (Jasus edwardsii) fishery in South Australia. (PDF) [file pone.0139816.s001.pdf]

SUPPORTING INFORMATION, S1 APPENDIX

SAMPLING SIZE

Predation risk within fishing gear and implications for South Australian rock lobster fisheries

Felipe Briceño, Adrian J. Linnane, Juan Carlos Quiroz, Caleb Gardner and Gretta T. Pecl

Table A provides information about sampling effort including mean and maximum pots sampled per day by year and fishing zone, including the variability among years by the coefficient of variation (CV%). Additionally, Table B provides information about sample size in relation to the total number of lobsters sampled by year and fishing zone in this study.

Table A: Mean and maximum number of pots sampled per day by year and zone used in this study to examine octopus depredation in the rock lobster (*Jasus edwardsii*) fishery in South Australia. The coefficient variation (CV %) is also included to reflect annual variation in numbers of pots sampled per day.

	Northern Zone			Southern Zone		
**Year**	**Mean pot/day**	**CV %**	**Max pot/day**	**Mean pot/day**	**CV %**	**Max pot/day**
1993	6.12	77	19	4.79	149	35
1994	3.38	88	16	3.31	138	35
1995	3.55	133	31	3.14	161	34
1996	4.98	76	19	6.31	130	41
1997	3.47	136	29	5.02	159	65
1998	3.23	131	33	3.53	144	33
1999	2.07	51	10	3.97	149	45
2000	1.94	42	5	2.12	38	4
2001	1.90	39	4	1.97	46	4
2002	1.96	38	4	2.16	102	21
2003	1.73	40	6	3.52	165	49
2004	2.16	73	10	4.58	150	41
2005	2.88	119	20	3.76	197	46
2006	2.58	59	11	3.45	174	49
2007	2.01	56	10	2.41	149	42
2008	2.02	99	14	3.06	169	36
2009	3.40	124	28	4.10	193	47
2010	6.94	110	34	4.18	192	55
2011	5.14	143	37	6.27	184	72
2012	3.25	116	20	6.58	205	72

Table B: Total lobsters sampled by year and fishing zone, including sex proportion, used in this study to examine octopus depredation in the rock lobster (*Jasus edwardsii*) fishery in South Australia.

	Northern Zone			Southern Zone			
**Year**	**Female**	**Male**	**Total**	**Female**	**Male**	**Total**	**Total size**
1993	56%	44%	458	59%	41%	469	927
1994	52%	48%	641	51%	49%	524	1165
1995	56%	44%	664	56%	44%	613	1277
1996	55%	45%	834	55%	45%	1300	2134
1997	53%	47%	604	54%	46%	1201	1805
1998	52%	48%	709	50%	50%	943	1652
1999	51%	49%	632	52%	48%	1692	2324
2000	49%	51%	826	53%	47%	1466	2292
2001	45%	55%	728	54%	46%	1606	2334
2002	52%	48%	606	49%	51%	939	1545
2003	51%	49%	436	54%	46%	1591	2027
2004	50%	50%	485	54%	46%	1850	2335
2005	50%	50%	614	53%	47%	1380	1994
2006	49%	51%	712	52%	48%	2010	2722
2007	50%	50%	560	55%	45%	1866	2426
2008	53%	47%	323	56%	44%	1403	1726
2009	50%	50%	654	51%	49%	1605	2259
2010	50%	50%	944	52%	48%	1297	2241
2011	48%	52%	984	54%	46%	1749	2733
2012	52%	48%	483	56%	44%	1443	1926
