# Supplementary material for: Predation Risk within Fishing Gear and Implications for South Australian Rock Lobster Fisheries
Source: PLoS One. 2015 Oct 21;10(10):e0139816. doi: 10.1371/journal.pone.0139816 (PMC4619570; doi:10.1371/journal.pone.0139816)
Supplement: S2 Appendix — Table A: Model candidates proposed to test dependency of size, sex and zone in lobster mortality by octopus depredation within rock lobster fishery (Jasus edwardsii) in South Australia. Table B: Model candidates proposed to test dependency of size, sex, MFA and lobster catch per unit effort (cpue) in lobster mortality by octopus depredation within rock lobster fishery (Jasus edwardsii) in South Australia. Figure A: Model selection criteria based on (a) Log-likehood (b) Akaike information criteria (AIC) used to define Model 1. Figure B: Model selection criteria based on (a) Log-likehood (b) Akaike information criteria (AIC) used to define Model 2. (PDF) [file pone.0139816.s002.pdf]

SUPPORTING INFORMATION, S2 APPENDIX

MODEL SELECTION

Predation risk within fishing gear and implications for South Australian rock lobster fisheries

Felipe Briceño, Adrian J. Linnane, Juan Carlos Quiroz, Caleb Gardner and Gretta T. Pecl

1.1 Testing dependency of life history traits and fishing zones (Model 1)

Six model candidates were considered (Table A). The first step was the selection of fixed factors, and then the random factors. Model selection was performed considering the lowest log-likelihood value (LogLike) and Akaike information criteria (AIC), considering also degree of freedom (df). Two models (M1 and M6) were selected considering a balance between criteria used (Figure A). Despite that M3 had the lowest AIC and LogLike we selected M6 because it includes the zone as a nesting process to avoid high variability among fishing zones. By using random effects we can constraint the temporal variation (eg year) by zone.

Table A: Model candidates proposed to test dependency of size, sex and zone in lobster mortality by octopus depredation within rock lobster fishery (*Jasus edwardsii*) in South Australia. Selected model is highlighted in grey.

LogLike	AIC	df	Model ID	Fixed effects	Random effects
-7541.357	15092.71	5	M1	size + sex + zone	year
-7537.845	15087.69	6	M2	size : sex + zone	year
-7538.507	15089.01	6	M3	size : sex + zone	size(year) – 1
-7536.497	15088.99	8	M4	size : sex + zone	year(zone) + year(size)
NC	NC	9	M5	size + sex + zone	size + year(zone)
**-7540.039**	**15092.08**	**6**	**M6**	**size + sex + zone**	**year(zone)**

NC: No convergence achieved

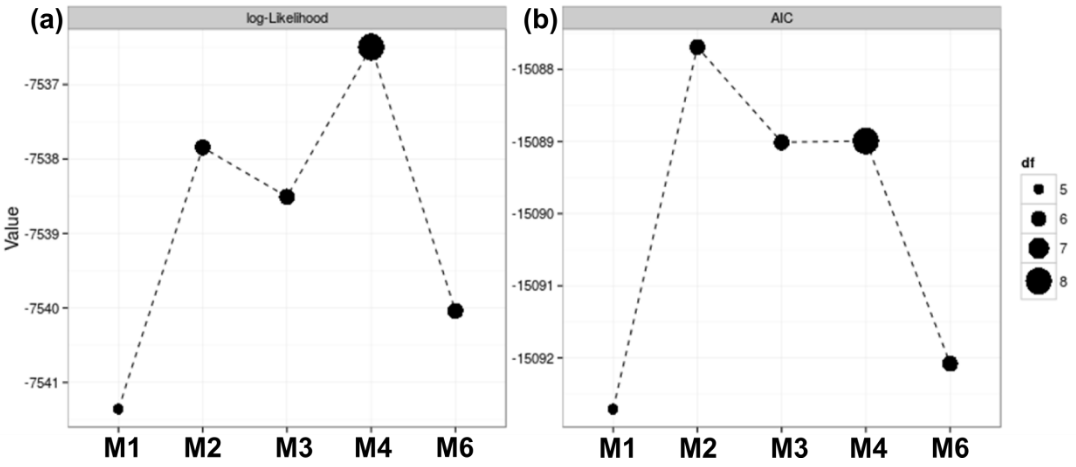

Figure A: Model selection criteria based on (a) Log-likehood (b) Akaike information criteria (AIC) used to define Model 1. Point size represents degree freedom (df). M5 is not included given no convergence was achieved.

1.2 Testing density-dependency with lobster catches (Model 2)

Five models candidates were used (Table B), considering the same criteria than in model 1. Three models accounted for such criteria (M1, M2 and M5) (Figure B). Given that lobster catch rates strongly vary across fishing season (month), we chose M2 because it includes such temporal variation to examine octopus depredation.

Table B: Model candidates proposed to test dependency of size, sex, MFA and lobster catch per unit effort (cpue) in lobster mortality by octopus depredation within rock lobster fishery (*Jasus edwardsii*) in South Australia. Selected model is highlighted in grey.

LogLike	AIC	df	Model ID	type	Fixed effects	Random effects
-5311.733	-10653.47	15	M1	GLMM	size + sex + cpue + MFA	Year + month
**-5312.515**	**-10655.03**	**15**	**M2**	**GLMM**	**size + sex + cpue:MFA**	**Year + month**
-5295.437	-10656.87	33	M3	GLMM	size + sex + cpue + MFA + year	month
-5285.767	-10649.53	39	M4	GLM	size + sex + cpue + MFA + year + month	--
-5312.182	-10668.36	22	M5	GLMM	sex + cpue + MFA + month	size(year)

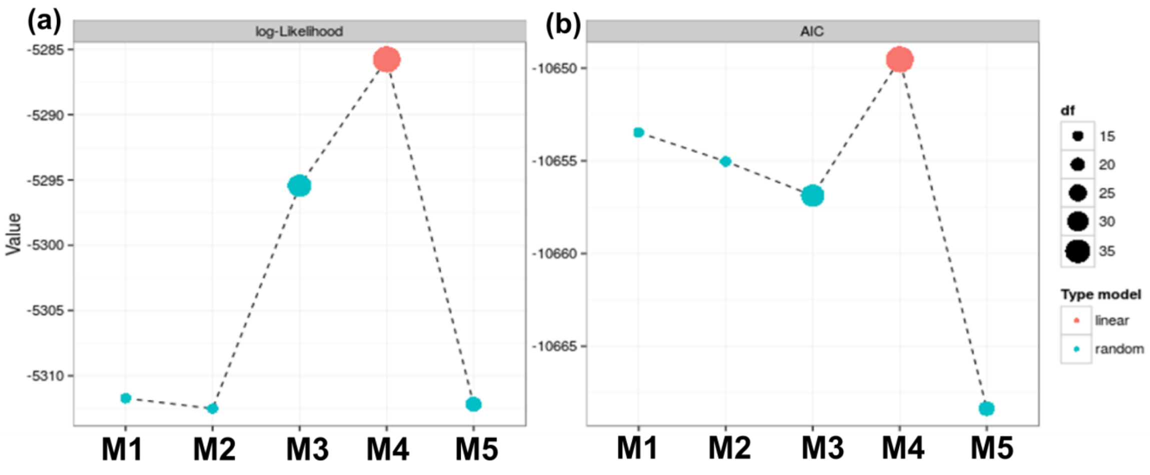

Figure B: Model selection criteria based on (a) Log-likehood (b) Akaike information criteria (AIC) used to define Model 2. Point sizes represent degree freedom (df). Colour show different models such as GLMM (blue) and GLM (red) used.
