# Supplementary figures and images for: Predation Risk within Fishing Gear and Implications for South Australian Rock Lobster Fisheries
Source: PLoS One. 2015 Oct 21;10(10):e0139816. doi: 10.1371/journal.pone.0139816 (PMC4619570; doi:10.1371/journal.pone.0139816)

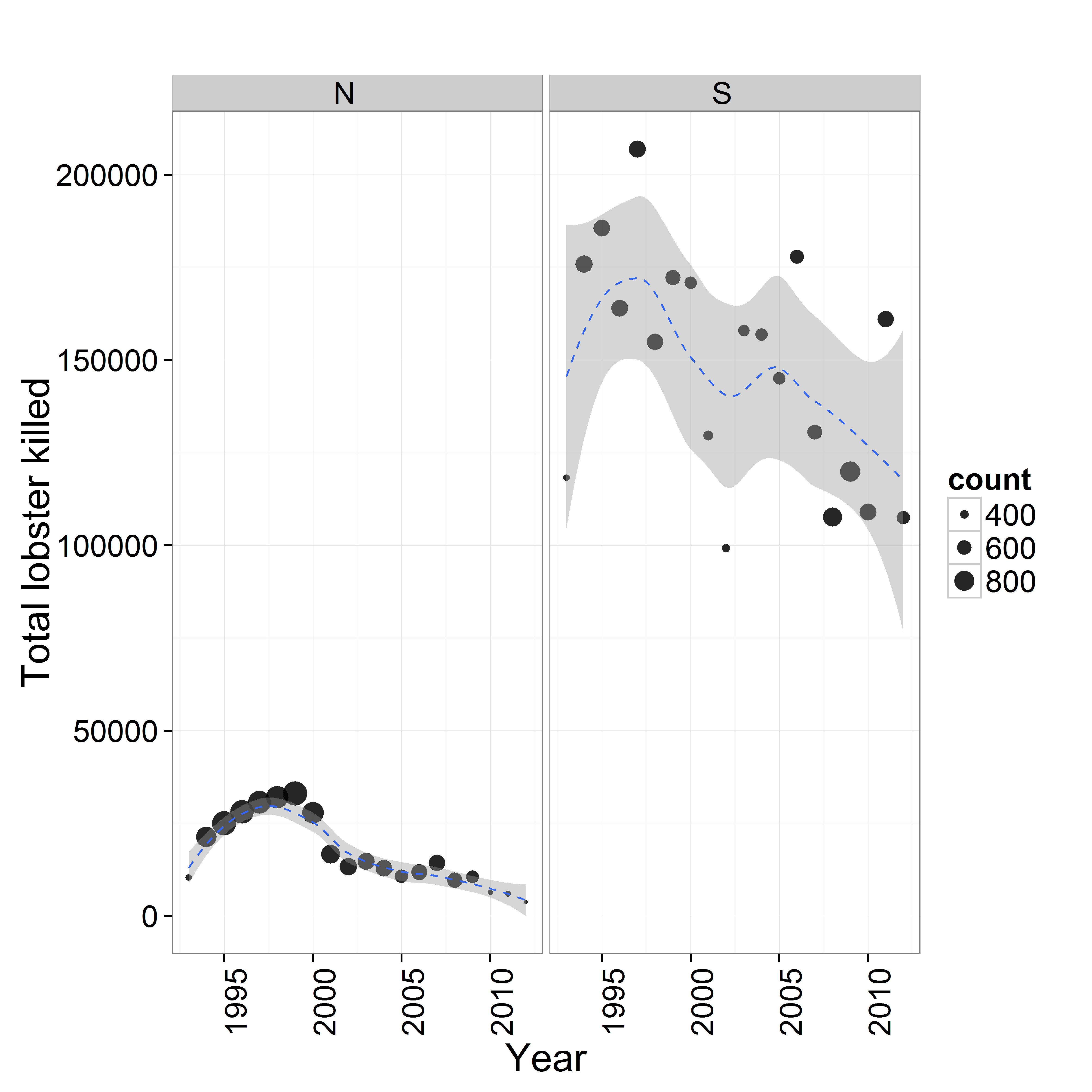

Supplement: S1 Fig — Dashed blue black lines represent the smoothing (polynomial), and the grey bands represent the confidence interval around the mean (sum+ 1.96*sd). (TIF) [file pone.0139816.s003.tif]

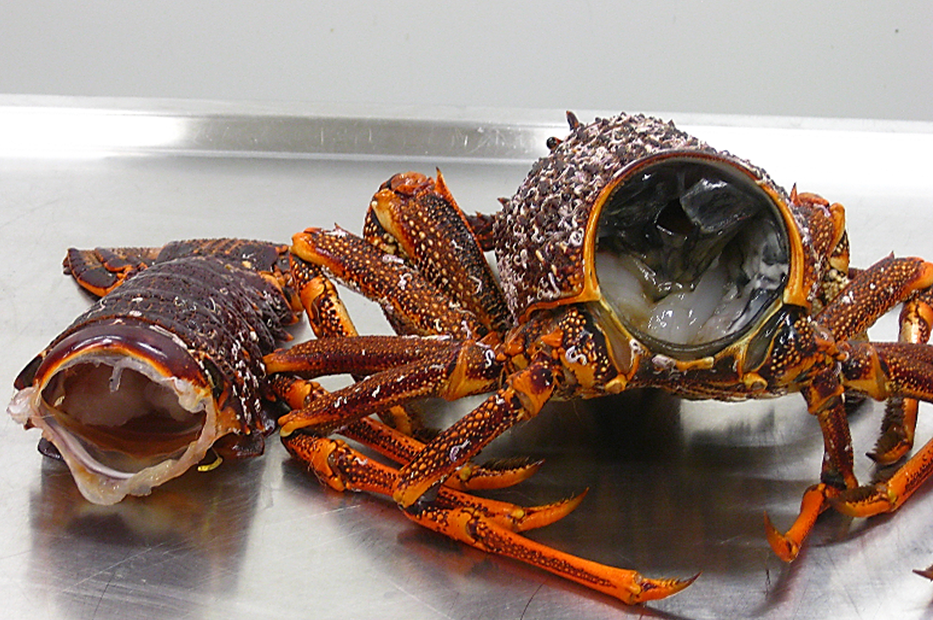

Supplement: S2 Fig — The picture shows the characteristic pattern of most of the muscle and viscera removed by the octopus (Felipe Briceño, February 2012). (TIF) [file pone.0139816.s004.tif]
